# Notch signaling mutations increase intra-tumor chemokine expression and predict response to immunotherapy in colorectal cancer

**DOI:** 10.1186/s12885-022-10032-5

**Published:** 2022-08-29

**Authors:** Fei Wang, Chuan Huang, Jie Long, Zhi-Bin Zhao, Hai-Qing Ma, Xue-Qing Yao, Liang Li, Zhe-Xiong Lian

**Affiliations:** 1 Guangdong Cardiovascular Institute, Guangdong Provincial People’s Hospital, Guangdong Academy of Medical Sciences, Guangzhou, 510080 Guangdong China; 2 Department of Oncology, Guangdong Provincial People’s Hospital, Guangdong Academy of Medical Sciences, Guangzhou, 510080 Guangdong China; 3 School of Medicine, South China University of Technology, Guangzhou, 510006 Guangdong China; 4 Medical Research Center, Guangdong Provincial People’s Hospital, Guangdong Academy of Medical Sciences, Guangzhou, 510080 Guangdong China; 5 Department of General Surgery, Guangdong Provincial People’s Hospital, Guangdong Academy of Medical Sciences, Guangzhou, 510080 Guangdong China; 6 Guangdong Provincial People’s Hospital Ganzhou Hospital, Ganzhou Municipal Hospital, Ganzhou, 341000 China; 7 Guangdong Provincial People’s Hospital, Guangdong Academy of Medical Sciences, Guangzhou, 510080 Guangdong China

**Keywords:** Colorectal cancer, Notch signaling, Mutation, Immunotherapy, Chemokine

## Abstract

**Background:**

The Notch signaling mutation is associated with enhanced anti-tumor immune response in colorectal cancer (CRC). In this study, we aim to investigate the underlying mechanism and the predictive potential of Notch signaling mutation for responding to immunotherapy in CRC.

**Methods:**

We analyzed the immune response associated genes in CRC with Notch signaling mutation concomitant with or without microsatellite instability (MSI) using TCGA dataset and investigated the mutation profiles of the Notch signaling pathway using cBioPortal. The Notch signaling scores and immune cell infiltration scores in different groups were calculated. We applied the Kaplan–Meier method for survival analysis in CRC patients who underwent immunotherapy, and the log-rank test to determine the statistically significant differences in survival. Notch1-knock-down cell line was constructed to detect the pathway and gene variations.

**Results:**

We found that Notch signaling pathway mutation was associated with activated immune response, especially in those with MSI. Such association is useful for predicting a prolonged overall survival of CRC patients who underwent immune checkpoint inhibitor treatment. The mutation resulted in the functional loss of Notch signaling and may modulate the tumor immune microenvironment by increasing the expression of chemokines that are important for recruiting immune cells.

**Conclusions:**

The Notch signaling mutation can modulate the chemotaxis of immune cells by upregulating the chemokine levels of the tumor immune microenvironment, and CRC patients with Notch signaling pathway mutation have better overall survival after immune checkpoint inhibitor treatment.

**Supplementary Information:**

The online version contains supplementary material available at 10.1186/s12885-022-10032-5.

## Background

Immune checkpoint inhibitors (ICIs) have great potential in prolonging the overall survival of patients in various kinds of cancers. However, a significant population of patients have only weak or no responses to ICIs [1, 2]. It has been reported that the higher tumor mutation burden (TMB), the more favorable response to ICIs [3–5]. In colorectal cancer (CRC), one of the most malignant tumors that results in greatly cancer-associated mortality in the world [6], ICIs receive better responses in deficient mismatch repair (dMMR) or microsatellite instability (MSI) tumors, which are associated with higher TMB [7], suggesting that the interplay between gene mutations and tumor immune microenvironment (TIME) can predict response to ICIs treatment.

The Notch signaling pathway is highly conserved and plays an important role in cell fate decisions [8–10]. The canonical Notch signaling cascade is composed of four receptors (Notch 1–4) and five ligands (Jagged 1, Jagged 2, Delta-like ligand 1 (DLL1), DLL3 and DLL4), while the non-canonical cascade is activated by the interaction of Notch receptors with other pathways such as the nuclear factor-κB (NF-κB) pathway and transforming growth factor-β (TGF-β) pathway [11, 12]. Several reports show that the Notch signaling pathway can either promote tumor progression by modulating epithelial-mesenchymal transition (EMT) or inhibit tumor growth by regulating the anti-tumor immune responses [13, 14]. There have been plenty of studies reporting the molecular mechanisms regarding the association between Notch signaling and immunotherapy responses. For example, inhibiting the Notch signaling can promote cytotoxicity of CD8^+^ T cells and enhance the secretion of proinflammatory cytokines such as IFN-γ, TNF-α, and IL-1β in CRC [15]. In addition, the Notch signaling can participate in inducing M1 versus M2 polarization of macrophages, showing great potential of anti-tumor therapy [16].

Mutation of Notch may be a predictor to favorable ICIs response in non-small cell lung cancer (NSCLC) [17, 18]. We previously found that the Notch signaling pathway mutations were associated with enhanced anti‐tumor immunity in CRC, and that gene sets correlated with activated immune responses were up-regulated in bladder cancer and cervical cancer patients with Notch mutations [19]. These results suggest that Notch mutation may be a potential marker to favorable ICIs responses in various kinds of tumors, not only in NSCLC.

In this study, we found that Notch signaling mutation was associated with activated immune responses of CRC, especially in those with MSI, and that it predicted a prolonged overall survival of CRC patients with ICIs treatment. The mutation resulted in the functional loss of Notch signaling and influenced the TIME by modulating the secretion of chemokines.

## Methods

### Cell line

The MC38 cell line derived from C57BL/6 murine colon adenocarcinoma cells was obtained from Professor Yangxin Fu of the University of Texas, Southwestern Medical Center. The HEK293T cell line was obtained from Professor Ping Gao of South China University of Technology. These two cell lines were both cultured in DMEM medium (SH30243.FS, HyClone, USA) containing 10% fetal bovine serum (FBS) (FND500, ExCell Bio, China) at 37 °C in a 5% CO_2_ atmosphere. All cells used in this study were mycoplasma-free.

### *Notch1* knock down in CRC cell line

Lentiviral vector plasmid containing short hairpin RNA (shRNA) targeting murine *Notch1* was obtained from Professor Ping Gao. The sequence of the anti-Notch1 shRNA was 5’-CCGGGCCCTTTGAGTCTTCATACATCTCGAGATGTATGAAGACTCAAAGG.

GCTTTTT-3’. The packaging plasmid pCMV-dR8.91, the envelope expressing plasmid VSVG and the lentiviral vector plasmids were transfected into HEK293T cells by PEI (23,966–2, Polysciences, USA). PLKO.1 vector was co-transfected as the control vector. MC38 cells were infected with 4 mL DMEM medium containing viruses and 8 μg/mL polybrene (H9268, Sigma, Germany) in 6 cm dishes. Puromycin (8 μg/mL, S7417, Selleck, China) was used to screen the cells containing the genome of lentiviruses.

### Real-time PCR

Total RNA was extracted from tumor cells by Trizol (15,596–026, Invitrogen, USA), and converted into cDNA by PrimeScript RT Reagent Kit (RR047A, Takara, Japan). RT-PCR was conducted using SYBR Premix Ex Taq II (RR820A, Takara, Japan), and the data were analyzed by LightCycle 96 software. The primer sequences were as follows:

Notch1:

Forward: GATGGCCTCAATGGGTACAAG (from 5’-3’).

Reverse: TCGTTGTTGTTGATGTCACAGT (from 5’-3’).

β-actin:

Forward: TTGCTGACAGGATGCAGAAG (from 5’-3’).

Reverse: ACATCTGCTGGAAGGTGGAC (from 5’-3’).

### Western blot

The total protein of tumor cells was extracted by RIPA buffer (89,901, Thermo Fisher Scientific), followed by the quantified procedure according to Thermo BCA kit (23,227, Thermo Fisher Scientific, USA). Total protein was separated by sodium dodecyl sulfate–polyacrylamide gel electrophoresis (SDS-PAGE) and then transferred to polyvinylidene fluoride (PVDF) membrane (Millipore, Germany). The membranes were blocked with 5% nonfat milk, followed by staining with primary antibodies. The primary antibodies included: rabbit anti-mouse Notch1 (ab52627, Abcam, 1:1000, UK) and rabbit anti-mouse GAPDH (AF1186, Beyotime, 1:5000, China). Secondary antibody was HRP-linked goat anti-rabbit IgG (SA00001-2, Proteintech, 1:2000, USA). Images were taken by Tanon-5200 Chemiluminescent Imaging System (Tanon, China).

### RNA sequencing and data analysis

Total RNA was extracted from 1 × 10^6^ MC38 cells stably transfected with control shRNA or shNotch1, and subjected to library construction and RNA sequencing using the NovaSeq6000 platform (Illumina, USA). Each group included three replicates. After being aligned to the mouse reference genome GRCm38 by STAR aligner, the raw FASTQ reads were screened for contamination. The matrix of the aligned reads was obtained by Picard RnaSeqMetrics and the FeatureCounts program was used to quantify the gene expression levels. The DESeq2 R package was applied to screen the differentially expressed genes. Genes with log_2_-fold change in mean expression greater than 0.5 and *p* value less than 0.05 were used for further analysis. Gene ontology (GO) analysis was performed to identify pathways that were up‐ and down‐regulated between the different groups using the R package cluster Profiler.

### Notch signaling pathway mutation profile analysis

We used 12 studies of CRC, a total of 4341 patients, to analyze the Notch signaling pathway mutation profile from the cBioPortal website (https://www.cbioportal.org/). The mutation profiles of *NOTCH1*, *NOTCH2*, *NOTCH3*, *NOTCH4* and *FBXW7* were further investigated.

### CRC TCGA dataset

The dataset we chose from the TCGA database includes 526 cases of CRC patients (https://gdc.cancer.gov/about-data/publications/pancanatlas), which was composed of transcriptome sequencing data, whole exon sequencing data, the pathological, MSI information and prognostic information [20].

### Analysis of TCGA CRC dataset

Patients of CRC with mutations in *NOTCH1*, *NOTCH2*, *NOTCH3*, *NOTCH4*, *NUMB* and *FBXW7* were included into the Notch mutation group. The germline mutations were excluded. The mRNA expression data, which were generated by the Illumina HiSeq V2 platform, were presented as FPKM for further analysis. We compared the average expression of immune checkpoint molecules (*PDCD1*, *CD274* and *CTLA4*) and effector molecules (*IFNG*, *GZMH*, *GZMM*, *GZMA* and *GNLY*) in different groups. The *p* values were calculated by the One-way ANOVA test in IBM SPSS statistics 21 (IBM). The Notch signaling score and immune cell infiltration score were calculated with single-sample gene set enrichment analysis (ssGSEA) [21] using the gene sets from the MsIgDB database and Yasin et al. [21].

### Analysis of CRC single-cell RNA sequencing dataset

The FPKM expression matrix of GSE97693 dataset [22] was downloaded from GEO database (https://www.ncbi.nlm.nih.gov/geo). We analyzed the single-cell transcriptome sequencing results of patients with adenocarcinoma (CRC01) and mucinous adenocarcinoma (CRC02) from this dataset. We excluded the cells in which the number of unique genes was more than 6000 or less than 200. Only genes expressed in 5 or more cells were used for further analysis. At the end, we obtained a data matrix of 25,372 genes and 688 cells. The detailed procedure of data processing is reported previously [19]. The enriched score of Notch signaling pathway was calculated with the GSVA package (version 1.36.2), using the gene set from the MsIgDB database. Nonparametric rank-sum test was used to evaluate the differences.

### The dataset of CRC patients with immunotherapy

We searched for datasets of CRC patients undergoing immunotherapy, which contained the data of gene mutation, from the PubMed database using Colorectal Cancer and Immunotherapy as the key words. The datasets included fit the following criteria:(1) CRC patients of the dataset underwent immunotherapy; (2) gene mutation was detected in CRC patients; and (3) patients' clinical information and gene mutation information can be obtained. The exclusion criteria included: (1) reviews; (2) the data is not available; and (3) the mutation detected on circulating tumor DNA. For survival analysis, we used the Kaplan‐Meier method to generate survival curves, and the log‐rank test to determine the statistically significant differences in survival. The Cox proportional-hazards model was applied to identify the risk factors for survival. The hazard ratio (HR) was calculated.

### Statistical analysis

Measurement data was present as mean ± standard deviation (SD). The expression of different markers among more than two groups were compared by One-way ANOVA test. Pearson correlation analysis was used to analyze the correlation coefficient and *p* value between NOTCH signaling score and immune effector molecules. T test was used to analyze the statistical differences of gene expressions between the control group and the knock-down group. The above analysis was performed using SPSS 21 (IBM, USA) or GraphPad Prism 8. Two-tailed *p* < 0.05 was considered statistically significant.

## Results

### Notch signaling mutation can activate immune responses in CRC, especially in those with MSI

**Fig. 1:**
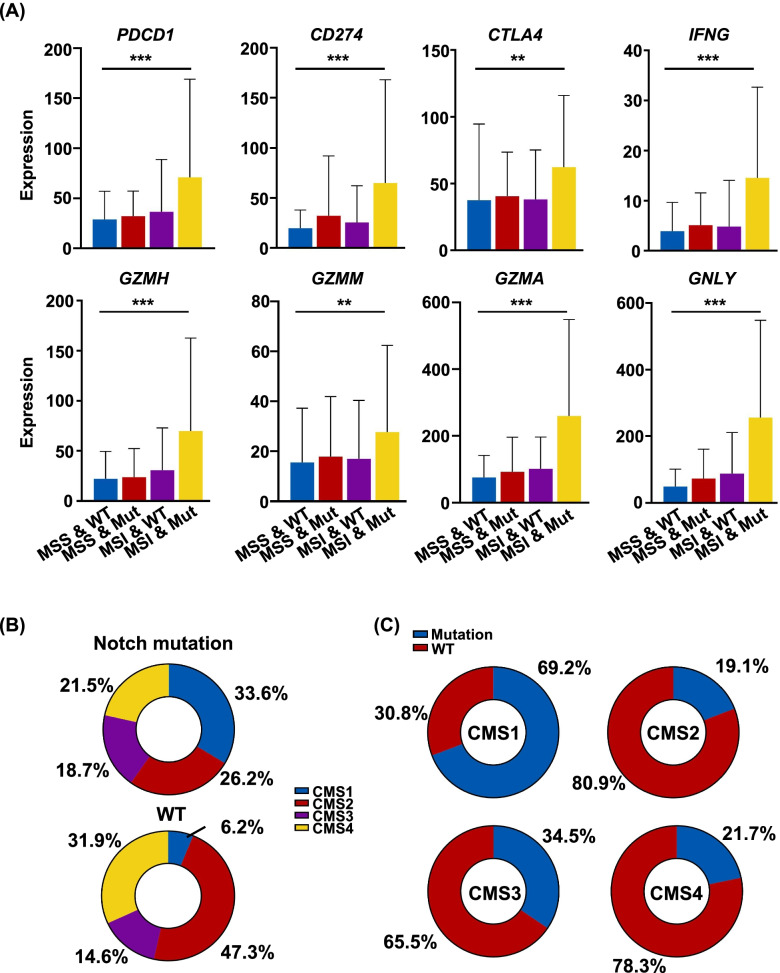
Notch signaling mutation activates immune responses in CRC. **A** The expressions of checkpoint and effector molecules in different groups of CRC patients in TCGA (*P* values were determined by the One-way ANOVA test. **p < 0.01, ***p < 0.001). **B** Proportions of patients with different CMS in mutation and WT groups. **C** Proportions of Notch mutation and WT patients in different CMS groups

We previously found that the Notch signaling pathway mutations were associated with activated anti-tumor immune response in CRC [19]. To further study the influence of Notch mutation on TIME in CRC, we divided CRC patients into four groups: MSI with or without mutation (MSI-WT & MSI-Mut) and microsatellite stable (MSS) with or without mutation (MSS-WT & MSS-Mut). We found the expressions of checkpoint molecules (*PDCD1*, *CD274*, *CTLA4*) and the effector molecules (*IFNG*, *GZMH*, *GZMM*, *GZMA*, *GNLY*) were the highest in the MSI-Mut group (Fig. 1A). Moreover, the expression levels of the molecules above were slightly higher in the MSS-Mut group than those in the MSS-WT group (Fig. 1A).

CRC can be divided into four groups: consensus molecular subtype 1 (CMS1), CMS2, CMS3 and CMS4 according to the genomic and transcriptomic characteristics [23]. The CMS1 subtype correlated with MSI and was characterized by hypermutation, hypermethylation and strong infiltration of immune cells in the tumor microenvironment (TME) [24]. The Notch signaling mutation had higher proportion of CMS1 than the WT group (Fig. 1B). In addition, the highest proportion of Notch signaling pathway mutations was observed in the CMS1 group, 69.2% (Fig. 1C).

Altogether, the Notch signaling pathway mutation can activate anti-tumor immune response of CRC, especially in those with MSI, and is present more in the CMS1 group.

### The mutation profiles of the Notch signaling pathway in CRC

**Fig. 2:**
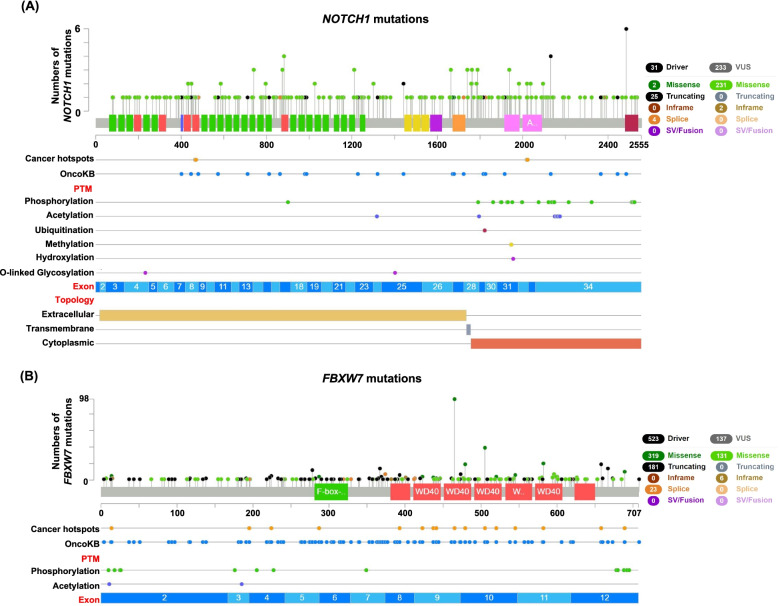
The mutation profiles of *NOTCH1* and *FBXW7* in CRC*.* **A** The mutation profile of *NOTCH1*. **B** The mutation profile of *FBXW7*. (PTM, post translational modification)

We further investigated the mutation profiles of the Notch signaling pathway in CRC. The Notch receptor is a trans-membrane protein including extracellular, transmembrane and cytoplasmic parts [25]. We found that most of the driver mutations of *NOTCH1* were truncating mutations, which often led to the amino acid (aa) loss of proteins. Mutations of the *NOTCH1* occurred more in the 700–1200 aa of the extracellular part and 1700–2100 aa of the cytoplasmic parts (Fig. 2A), and the later one included the ubiquitination site. The mutation profile of *NOTCH1* was similar with *NOTCH2*, *NOTCH3* and *NOTCH4* (Figure s1A, B and C). The FBXW7 is a critical component of the Notch signaling pathway, participating in the ubiquitination-degradation of the cytoplasmic part of notch receptor [26]. Most of the driver mutations of *FBXW7* were missense mutations (Fig. 2B). The truncating mutations occurred more often in the 0–400 aa of the *FBXW7*. However, the missense mutations occurred more often in the 400–707 aa (Fig. 2B).

### The Notch pathway mutations lead to the loss of function of Notch signaling

**Fig. 3:**
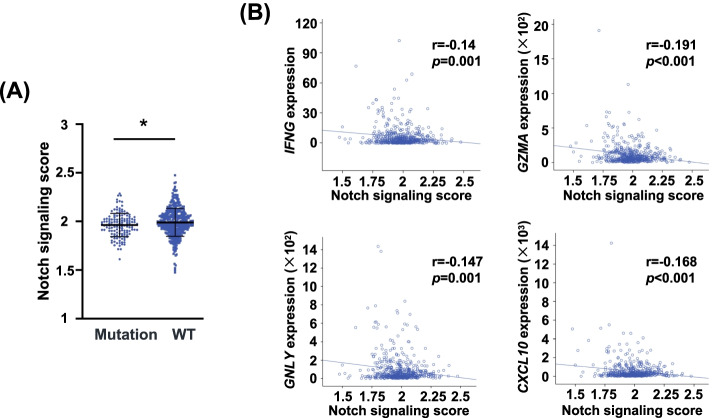
The correlations of Notch signaling score and immune-related molecules in CRC. **A** The Notch signaling score in the mutation and WT groups (*p < 0.05). **B** The correlations of Notch signaling score with *IFNG*, *GZMA*, *GNLY* and *CXCL10*

To explore whether the Notch signaling mutations lead to loss of function or gain of function, we firstly analyzed the Notch signaling score in CRC tumors with or without Notch mutation. We found that the mutation group had lower Notch signaling score than the WT group (*p* < 0.05) (Fig. 3A). We then analyzed the association between expression levels of effector molecules (*IFNG*, *GZMA*, *GNLY* and *CXCL10*) and Notch signaling score. Negative correlations between Notch signaling score and effector molecules (*IFNG*, *GZMA*, *GNLY* and *CXCL10*) were observed (Fig. 3B). Because of the activated immune response caused by Notch mutation, these results indicated that the Notch mutation may lead to the loss of function of this signaling pathway.

### Decreased Notch signaling in cancer cells from mucinous adenocarcinoma

**Fig. 4:**
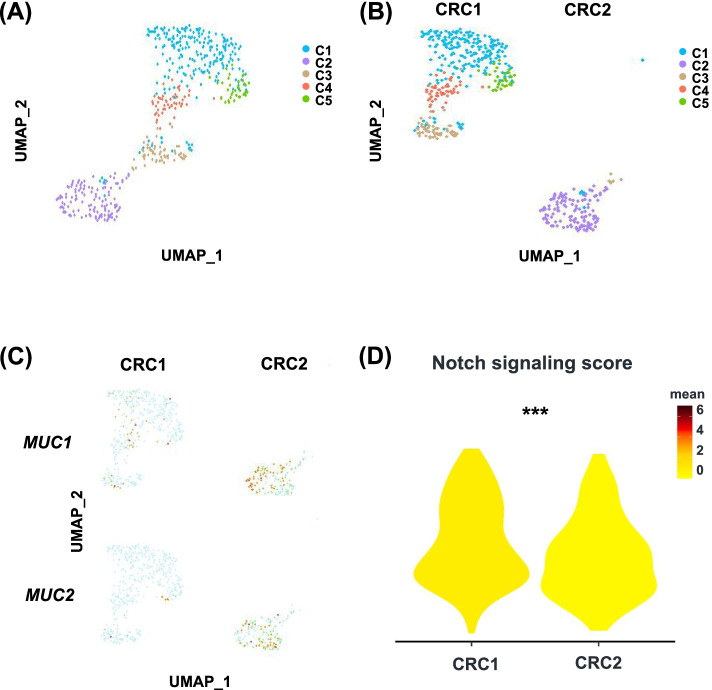
Notch signaling scores in adenocarcinoma and mucinous adenocarcinoma. **A** U-map visualization of tumor cell clusters from two CRC patients. **B** U-map visualization of CRC1 and CRC2 clusters. **C** Feature plots showing the expressions of *MUC1* and *MUC2* in CRC1 and CRC2 patient. **D** Violin plots showing the NOTCH signaling score in CRC1 and CRC2 patient

We previously found that CRC patients with Notch signaling mutation had higher proportion of mucinous adenocarcinoma [19]. Therefore, we hypothesized that the Notch signaling is associated with the differentiation status of tumor cells. We calculated the Notch signaling enrichment score of two patients from single-cell RNA sequencing dataset GSE97693. CRC1 is adenocarcinoma, and CRC2 is mucinous adenocarcinoma. Five cancer cell clusters were identified using unsupervised clustering (Fig. 4A). CRC1 and CRC2 had distinct cancer cell distribution profiles (Fig. 4B). Most cells in C2 were from CRC2. The mucinous adenocarcinoma characterized genes, such as *MUC1* and *MUC2*, were significantly enriched in cancer cells from CRC2 (Fig. 4C). Consistent with what we have hypothesized, CRC1 had higher Notch signaling score than CRC2 (Fig. 4D). In the normal intestinal epithelium, the Notch signaling pathway can promote the stem cell to differentiate into the enterocyte and inhibit the development of secretary cells, including the goblet cells [27, 28]. Our results suggested that the Notch signaling can not only modulate the differentiation of normal intestinal epithelium, but also influence the differentiation of CRC.

### The Notch signaling mutation modulates the TIME by upregulating the chemotaxis of immune cells

**Fig. 5:**
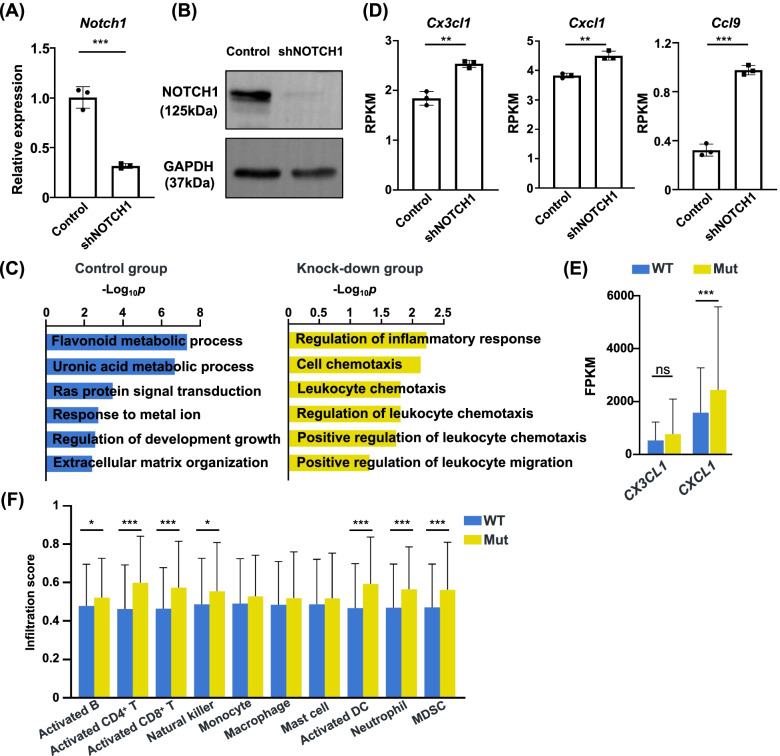
Deficiency of Notch signaling leads to increased chemokine expression in CRC tumor microenvironment. **A** RT-PCR result showing decreased *NOTCH1* expression in MC38 cell line stably transected with shNOTCH1 (***p < 0.001). **B** WB result showing decreased NOTCH1 protein level in MC38 cell line stably transected with shNOTCH1. Full-length blot is presented in the supplementary material. **C** Gene sets enriched in the control and knock-down groups. **D** The expressions of *Cx3cl1*, *Cxcl1* and *Ccl9* in the control and knock-down groups (**p < 0.01, ***p < 0.001). **E** The expression levels of *CX3CL1* and *CXCL1* in the WT and mutation patients of TCGA (ns, not significant, ***p < 0.001). **F** The infiltration scores of different immune cells in the WT and mutation patients of CRC in TCGA (*p < 0.05, ***p < 0.001)

To investigate the possible mechanisms for the activated immune response of Notch signaling mutation, we constructed MC38 cell line with *Notch1* knock-down. *Notch1* mRNA expression was significantly downregulated after infection with lentivirus carrying anti-*Notch1* shRNA (Fig. 5A). The WB result also showed the decrease of NOTCH1 protein (Fig. 5B). We further performed RNA-sequencing of control and MC38 cells with *Notch1* knock-down. GO analysis revealed that the chemokine related pathways were significantly enriched in the *Notch1* knock-down cells (Fig. 5C). We observed that the *Cx3cl1*, *Cxcl1* and *Ccl9* were significantly upregulated in the *Notch1* knock-down group (Fig. 5D). The expression levels of *CX3CL1* and *CXCL1* in Notch mutation patients were higher than those without mutation, especially CXCL1 (*p* < 0.001) (Fig. 5E). The infiltration scores of anti-tumor immune cells including activated B cells, activated CD4^+^ T cells, activated CD8^+^ T cells, natural killer cells, and activated dendritic cells (DC) increased in the mutation group (Fig. 5F). Interestingly, the pro-tumor immune cells including neutrophils and myeloid-derived suppressor cells (MDSC) were also significantly higher (Fig. 5F). These results indicated the loss-of-function mutation of the Notch signaling can upregulate the chemokine levels of the TIME, which can recruit both pro-tumor and anti-tumor immune cells.

### The Notch mutation is associated with improved prognosis of CRC patients underwent immunotherapy

**Fig. 6:**
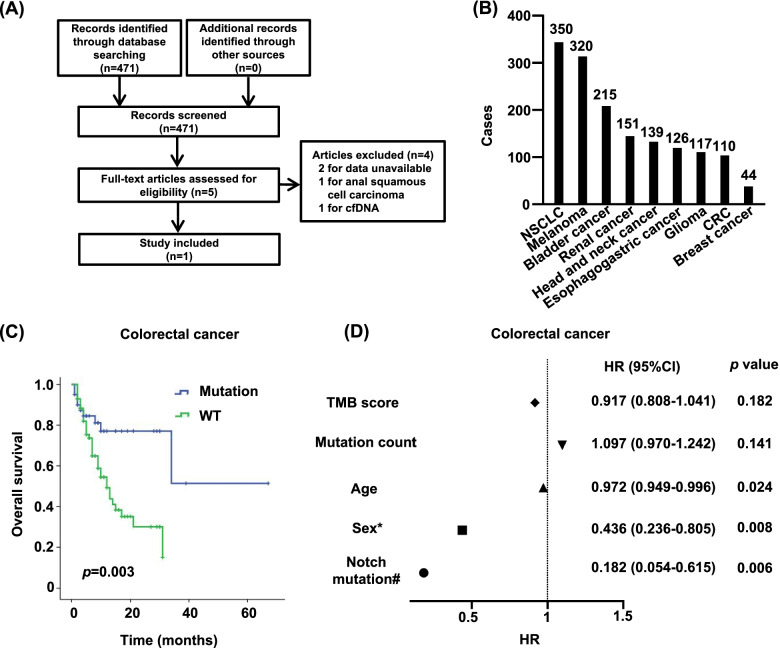
Survival analysis of CRC patients with immunotherapy. **A** Schematic overview of selecting articles involved in assessing the immunotherapy efficacy of CRC. **B** Cases in different cancers. **C** Overall survival of CRC patients with immunotherapy. **D** Forest plot showing the hazard ratio (HR) of each factor. *Female as the reference. #Non-mutation group as the reference. CI, confidence interval

Because of the upregulated expression levels of checkpoint and effector molecules in CRC patients with Notch mutation, we investigated the predictive value of Notch signaling mutation in immunotherapy response. We searched articles to evaluate the ICIs effect of CRC patients in the PubMed and identified 471 articles after initial searching (Fig. 6A). After evaluating and filtering, one study was included for further analysis [5] (Fig. 6A). Nine cancer types and 1572 cases for ICIs treatment were included in this article [5] (Fig. 6B). Most patients (1,446, representing 94% of tumors excluding glioma) had stage IV or metastatic disease. In this study, only the mutations of *NOTCH1*, *NOTCH2*, *NOTCH3*, *NOTCH4* and *FBXW7* were assessed. As we expected, the K-M survival curve showed that the CRC patients with Notch signaling mutation had significantly prolonged overall survival after ICI treatment (*p* = 0.003) (Fig. 6C). The Cox proportional-hazards model showed the Notch signaling pathway mutation played a protective role in the survival of CRC patients with immunotherapy (*p* = 0.006) (Fig. 6D). Meanwhile, the prolonged overall survival was also observed in the NSCLC patients with Notch mutation (*p* = 0.042) (Figure s2A). However, we did not observe any significant associations between the Notch mutation and the overall survival in other cancer types (Figure s2A). These results indicated that CRC patients with Notch signaling mutations may benefit from ICIs treatment.

## Discussion

The association between Notch mutation and immunotherapy has been reported in the NSCLC [17, 18]. In our previous study, we have found that the Notch signaling pathway mutations are associated with enhanced anti‐tumor immunity in CRC [19]. In this study, we further investigated the mechanism of the activated immune response after Notch mutation and the effect of Notch mutation on the efficacy of immunotherapy in CRC. Our results showed that Notch signaling mutation can modulate the chemotaxis of immune cells by upregulating the chemokine levels of the TIME and CRC patients with Notch signaling pathway mutation had better overall survival after ICIs treatment.

The mutation of Notch signaling may lead to the activation or inactivation of this pathway. In our study, we found that patients with mutations had lower level of Notching signaling score. Patients with lower level of Notch signaling score had higher levels of effector and checkpoint molecules. These results indicated the Notch signaling pathway mutation resulted in the loss of function of this signaling. In the normal state of the intestinal epithelium, the Notch signaling pathway can inhibit the differentiation of secretary cells, including goblet cell, which is the main cell type secreting mucus in the intestine [27, 28]. After carcinogenesis of the epithelial cells, the Notch signaling can also influence the pathology type of CRC, the mutation of which may lead to higher proportion of mucinous adenocarcinoma [19], a pathology type full of mucus in the tumor mesenchyme. This result also suggested the Notch signaling mutation may result in the loss of function of this pathway.

In our previous study, although higher proportion of early stage and anti-tumor response existed in the mutation group, no significant differences were observed in the disease-free survival and overall survival between the mutation and WT groups [19]. This may be caused by the higher proportion of mucinous adenocarcinoma in the mutation group [19]. Studies have showed that mucinous adenocarcinoma is an independent poor prognostic factor in CRC patients [29, 30]. However, due to the activated immune response in the TIME of mutation patients, ICIs treatment can improve the overall survival of mutation patients of CRC, which indicated the Notch signaling mutation can be a biomarker for predicting the efficacy of immunotherapy in CRC.

Although the Notch signaling mutation influences the TIME [17, 18], the mechanisms are still unclear. The gene set enrichment analysis showed pathways involved in the chemotaxis were upregulated in the NOTCH1-knock-down group. Chemokines, such as *CX3CL1*, *CXCL1* and *CCL9*, were significantly upregulated in the knock-down group. CX3CL1 can recruit the cells expressing CX3CR1, which is widely present on the immune cells, especially the monocyte/macrophage and DC. The CX3CR1^+^ DC in the intestine has been shown strong antigen uptake ability [31], contributing to the activation of adaptive immune response. The CX3CR1^+^ CD8^+^ T cells display an enhanced cytolytic function as compared to other CD8^+^ T cells [32] and can indicate response to ICI therapy [33, 34]. The CXCL1 can bind to the CXCR2 receptor, which is found on neutrophils, T lymphocytes, monocytes/macrophages and eosinophils [35]. The CCL9 also plays a critical role of recruiting immune cells into the tissues by interacting with CCR1 [36, 37]. In our study, we observed the increase of lymphoid cells, including CD4^+^ T cell, CD8^+^ T cells and B cells in the mutation patients, which may be caused by the upregulation of chemokines in the TME of mutation group. However, the infiltration of myeloid cells, such as DC, neutrophil and MDSC were also increased in the mutation group, which may lead to the exhaustion of CD8^+^ T cells in the mutation group. This may be the reason that Notch mutation is not associated with better overall survival of CRC patients, and patients with Notch signaling mutation can benefit from ICI therapy.

In summary, we investigated the association between the Notch mutation and the immunotherapeutic efficacy and the mechanism of activated anti-tumor immune response caused by Notch mutation. We found CRC patients with Notch mutation can benefit from immunotherapy. The upregulation of chemokines caused by the Notch mutation may contribute to activating the anti-tumor immune response of the TME in CRC patients. However, it is hard to assess the influence on immunotherapeutic efficacy of different mutation types or sites because of the limited sample size, which is worthy of further investigation.

## Conclusions

The Notch signaling mutation can modulate the chemotaxis of immune cells by upregulating the chemokine levels of the tumor immune microenvironment. CRC patients with Notch signaling pathway mutation have better overall survival after ICIs treatment.

## Supplementary Information

**Additional file 1: Figure s1.** The mutation profiles of *NOTCH2*, *NOTCH3* and *NOTCH4* in CRC. **Figure s2.** Survival analysis of other cancer patients with immunotherapy.

**Additional file 2. **

## Data Availability

The single tumor cell sequencing raw data are available as GEO accession GSE97693 at https://www.ncbi.nlm.nih.gov/geo/. Gene-expression data, whole exome sequencing data and clinicopathological data of TCGA cohort were downloaded from the UCSC Xena browser (GDC hub: https://gdc.xenahubs.net).

## Abbreviations

CRC: Colorectal cancer
CMS: Consensus molecular subtype
DC: Dendritic cell
dMMR: Deficient mismatch repair
EMT: Epithelial-mesenchymal transition
GO: Gene ontology
GSEA: Gene set enrichment analysis
ICI: Immune checkpoint inhibitor
MDSC: Myeloid-derived suppressor cell
MSI: Microsatellite instability
MSS: Microsatellite stable
NSCLC: Non-small cell lung cancer
TCGA: The Cancer Genome Atlas
TIME: Tumor immune microenvironment
TMB: Tumor mutation burden
TME: Tumor microenvironment
WB: Western Blot; WT: wild type
