# Supplementary material for: Notch signaling mutations increase intra-tumor chemokine expression and predict response to immunotherapy in colorectal cancer
Source: BMC Cancer. 2022 Aug 29;22:933. doi: 10.1186/s12885-022-10032-5 (PMC9426242; doi:10.1186/s12885-022-10032-5)
Supplement: Supplementary file 1 — Additional file 1: Figure s1. The mutation profiles of NOTCH2, NOTCH3 and NOTCH4 in CRC. Figure s2. Survival analysis of other cancer patients with immunotherapy. [file 12885_2022_10032_MOESM1_ESM.pdf]

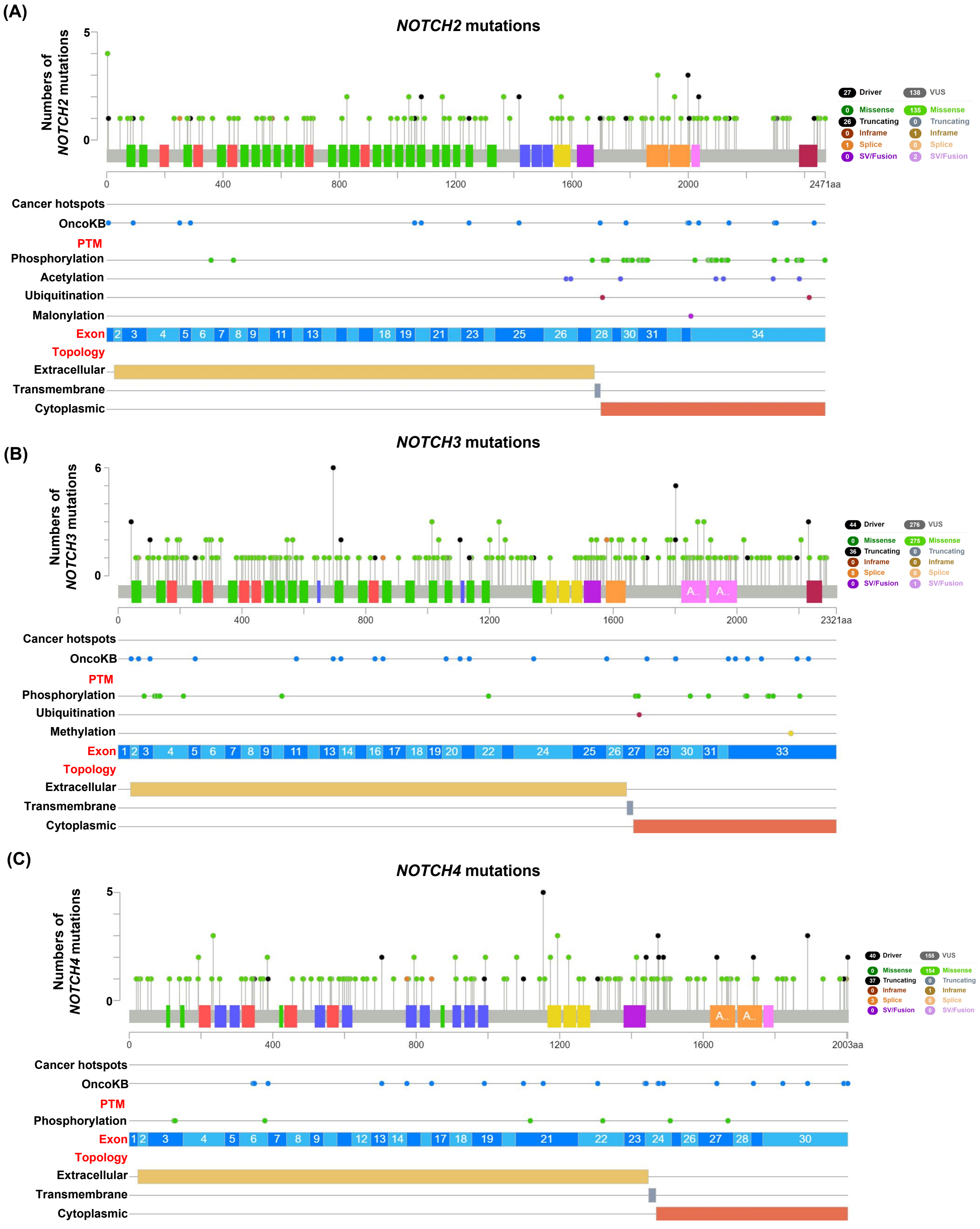

Figure s1 The mutation profiles of *NOTCH2, NOTCH3* and *NOTCH4* in CRC

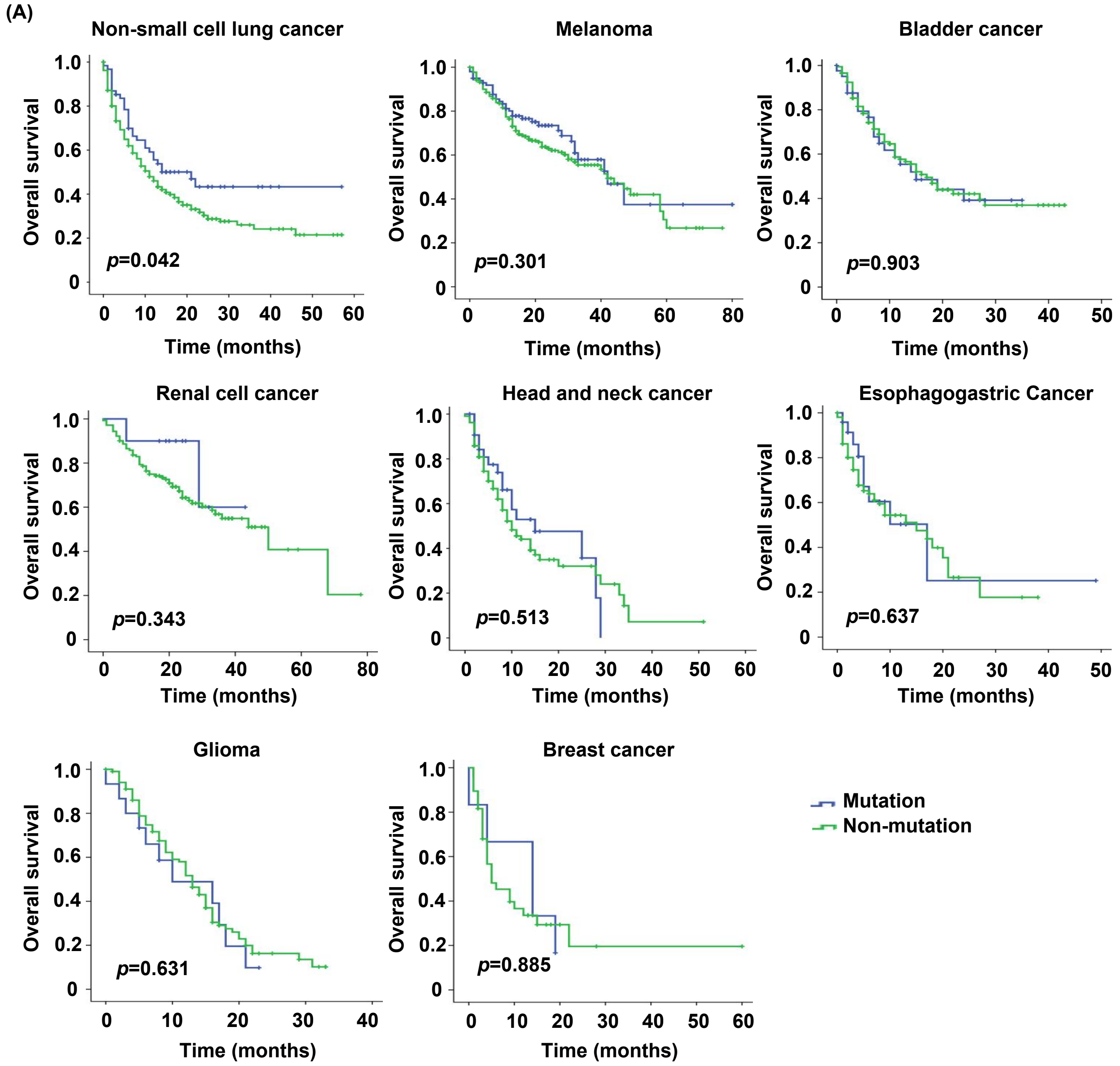

Figure s2 Survival analysis of other cancer patients with immunotherapy

Figure s1 The mutation profiles of *NOTCH2*, *NOTCH3* and *NOTCH4* in CRC.
(A) The mutation profile of *NOTCH2*. (B) The mutation profile of *NOTCH3*. (C) The mutation profile of *NOTCH4*. (PTM, post translational modification)

Figure s2 Survival analysis of other cancer patients with immunotherapy.
(A) Overall survival of cancer patients with immunotherapy.
