# Supplementary figures and images for: Notch signaling mutations increase intra-tumor chemokine expression and predict response to immunotherapy in colorectal cancer
Source: BMC Cancer. 2022 Aug 29;22:933. doi: 10.1186/s12885-022-10032-5 (PMC9426242; doi:10.1186/s12885-022-10032-5)

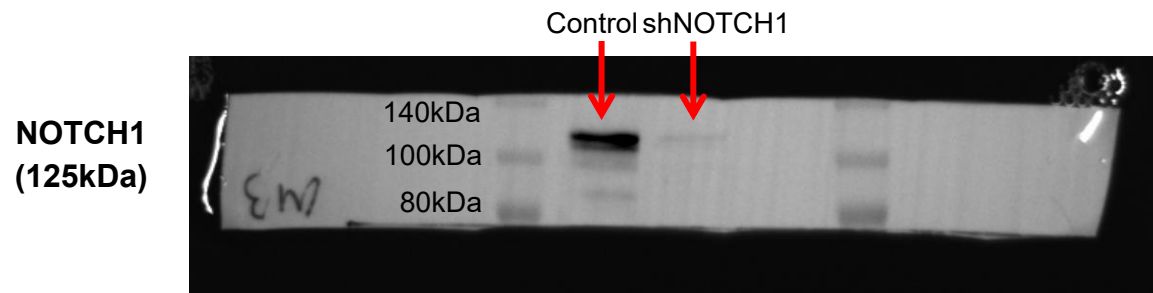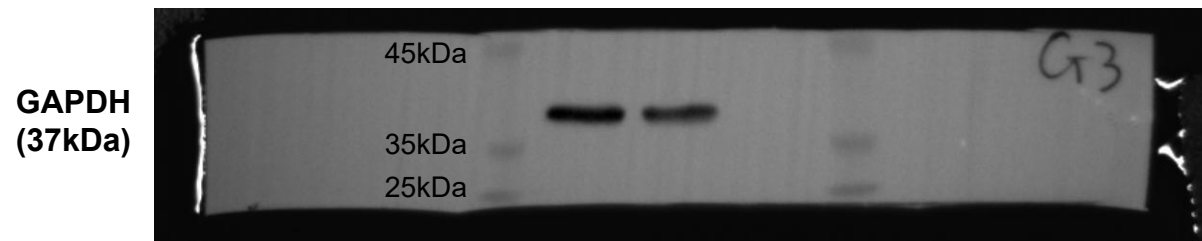

Supplement: Supplementary file 2 — Additional file 2. [file 12885_2022_10032_MOESM2_ESM.pdf]
